# Genetic Diversity and Population Structure of Llamas (*Lama glama*) from the Camelid Germplasm Bank—Quimsachata

**DOI:** 10.3390/genes11050541

**Published:** 2020-05-12

**Authors:** Gabriela F. Paredes, Claudia E. Yalta-Macedo, Gustavo A. Gutierrez, Eudosio A. Veli-Rivera

**Affiliations:** 1 Laboratorio de Biología Molecular y Genómica, Dirección de Recursos Genéticos y Biotecnología, Instituto Nacional de Innovación Agraria (INIA). Av. La Molina 1981, 15024 Lima, Peru; cyalta@inia.gob.pe; 2 Facultad de Zootecnia, Universidad Nacional Agraria La Molina (UNALM), Av. La Molina s/n, 15024 Lima, Peru; gustavogr@lamolina.edu.pe

**Keywords:** *Lama glama*, Camelid Germplasm Bank—Quimsachata, microsatellites, genetic diversity, population structure

## Abstract

Llamas (*Lama glama*) are invaluable resources of Peru. Despite their importance, their population is decreasing. The Camelid Germplasm Bank—Quimsachata was created as a guardian of this South American camelid (SAC) species and established a bank of llamas from their two types, Ch’aku and Q’ara. However, these populations need to present high genetic diversity to be considered suitable conservation stocks. Thus, in the present study, 13 microsatellites specific for the SAC were used to assess the current genetic variability and differentiation of the llama population from the Bank. The global population showed high genetic diversity with a total of 157 different alleles, with an average of 12.08 alleles per microsatellite, an expected and observed heterozygosity of 0.758 and 0.707, respectively, and an average polymorphic information content (PIC) of 0.723. Although considered as two different breeds and managed separately, the genetic differentiation between Ch’aku and Q’ara was low (F_ST_ = 0.01). Accordingly, the gene flow value was high (Nm = 30.5). Overall, our results indicate the existence of high genetic variation among individuals, and thus, this llama population could be considered a suitable genetic stock for their conservation and for sustainability programs. Additionally, the 13 microsatellites can be used to study other Peruvian llama populations and monitor the genetic variability of llamas from the Camelid Germplasm Bank—Quimsachata.

## 1. Introduction

Llamas (*Lama glama*) are the largest South American camelids (SACs) and the best adapted to a wide range of environmental conditions [1]. They can be mainly found in the Andean region between 2300 and 4000 m above sea level in Peru, Bolivia, Ecuador, northwest Argentina, and central Chile [2]. After Bolivia, Peru is the country with the second-largest population of llamas in the world [3], being primarily found in the Department of Puno, with almost 35% of the total Peruvian llama population [4]. For the local economy, llamas are seen as multipurpose animals; given their low-fat and low-cholesterol but high-protein content, their meat is consumed [2], and occasionally their intestines are used to make string and drums, while their excrement is used as fuel. Moreover, they are pack animals and their fiber is often used for clothing [5]. Two main types of llamas are recognized: Q’ara and Ch’aku (or T’hampulli). The Q’ara is a light-wool type with a long and slim body and has lower quality fiber, but it possesses a greater aptitude for meat production [6,7,8]; the heavy-wool type, Ch’aku, has a shorter body but has a higher potential for fiber production [9]. 

Despite being used for different utilities, the population of llamas is decreasing, with around 300,000 fewer llamas reported in the last national census [10] compared to the one in 1994 [11,12]. The diminishing llama population in Peru is due to various factors, such as the intense selection of the white fiber of alpacas, which leads to the decrease of colored (non-white) alpacas and llamas in order to increase the rearing of more white alpacas [13]. Furthermore, llama keepers tend to be neglected, as project funding greatly benefits the production system of other SAC species, mainly alpacas [12,14].

Therefore, before the imminent loss of their phenotypic and genetic diversity, in 1987 the National Institute of Agricultural Innovation (INIA) established the “Camelid Germplasm Bank—Quimsachata” in Puno, Peru, oriented to the recovery and conservation of colored alpacas and the two llama types, Ch’aku and Q’ara [13]. However, to be considered adequate genetic stocks, these populations must present high genetic diversity to guarantee the conservation and augment the variability and productivity of these animals [15]. However, to date, there are no genetic characterization studies of the llama population from the Bank that could help to develop appropriate management strategies, detect rare alleles that indicate the presence of unique genetic variants [16], and avoid inbreeding depression [17].

Microsatellite markers (hereafter referred to as microsatellites), due to their high polymorphism and abundant distribution throughout the genome [18], are important tools for the assessment of genetic diversity and the structure of livestock populations [19,20,21]. There are over 70 nuclear microsatellites reported for the SAC [22,23,24,25,26,27,28,29] that can be potentially used for the genetic characterization of SAC populations—a first step in making conservation strategies. In Peru, most studies (some involving microsatellites) have been carried out in alpaca and vicuña populations [21,30,31,32], whereas in llama populations these were either focused on studying their origin [29,33,34] or on analyzing quantitative parameters related to the physiology, production, growth, or fiber of the animal [35,36,37,38].

Therefore, this is the first population genetics study employing microsatellites carried out on the llama population of the Camelid Germplasm Bank—Quimsachata, whose biodiversity represents the natural, economic, cultural, and historical patrimony of Peru, and it aims to (i) assess its genetic diversity and structure and (ii) estimate the genetic differentiation between the two llama subpopulations, Ch’aku and Q’ara.

## 2. Materials and Methods 

### 2.1. Sample Collection

**Figure 1:**
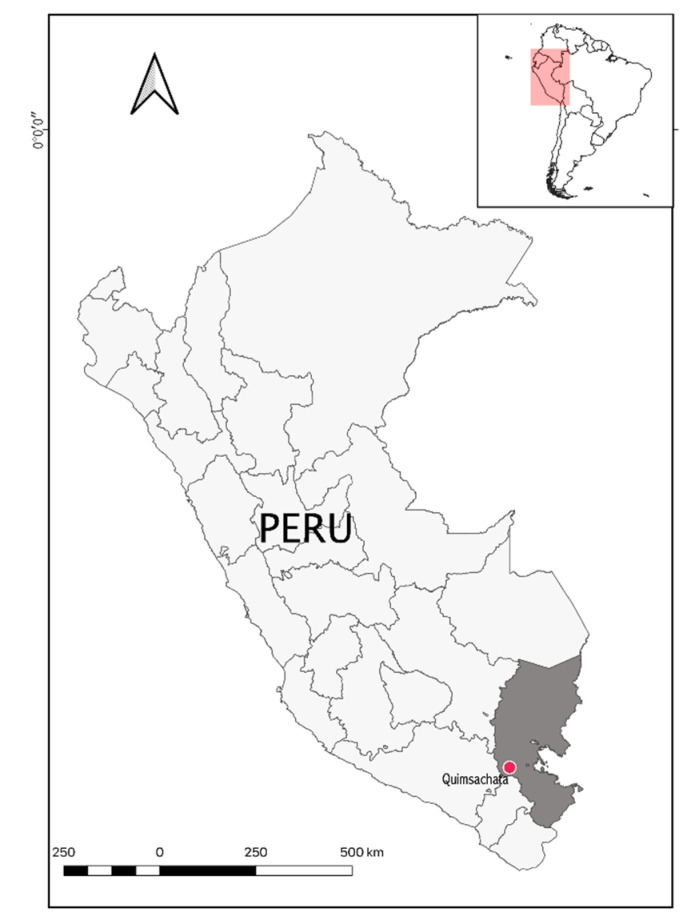
Geographical localization of the Camelid Germplasm Bank—Quimsachata in the Department of Puno, Peru at around 4200 m above sea level (map created with DIVA-GIS software).

Blood samples from 251 adult llamas of types Ch’aku (*n* = 92, 67 females and 25 males) and Q’ara (*n* = 159, 109 females and 50 males) were collected at the Camelid Germplasm Bank—Quimsachata, located in the district of Santa Lucia in the Department of Puno, Peru (Figure 1). The protocol used for the blood sample collection agreed with the requirements of the National Law No. 30407 “Ley de Protección y Bienestar Animal (Animal Protection and Welfare Law)”. To measure the genetic differentiation between both phenotypes, Ch’aku and Q’ara llamas were considered as two different subpopulations. Llamas were selected after analyzing their pedigree records, and only unrelated animals were sampled. The difference in the number of animals between the two populations and in the number of males and females that were enrolled in the study were due to the fact that the Camelid Germplasm Bank—Quimsachata has more Q’ara than Ch’aku phenotypes and a male to female ratio of approximately 30 to 70. Thus, the sampling was designed to keep this ratio and to have a representative sample of the population. Total genomic DNA was extracted using a standard phenol–chloroform and ethanol precipitation protocol [39]. The DNA pellet was resuspended in TE buffer and stored at −20 °C.

### 2.2. DNA Amplification and Microsatellite Genotyping

Thirteen microsatellites specific to llamas and alpacas, namely LCA82, LCA54, LCA65, LCA83, LCA77, LCA85, YWLL08, YWLL44, YWLL59, LAB1, GLM4, Lgu76, and VOLP03 (Table S1), were chosen due to their high polymorphic information content (PIC), elevated heterozygosity, and genetic diversity [22,24,25,26,28,40] and were genotyped across all samples. Genotyping was performed following the procedure described by De Arruda et al. (2010) [41]. The forward primer carried an extension sequence of 19 bp attached to its 5′ end (M13 sequence), which allowed labeling with three different fluorescent dyes (6-FAM, NED, and HEX; Table S1). The reverse primer remained unaltered.

Genomic DNA was amplified by polymerase chain reaction (PCR) using a Mastercycler Pro S (Eppendorf, Hauppauge, NY, USA). Microsatellites with similar PCR conditions were co-amplified using a multiplex PCR, and the fluorescent labeling of the forward primers allowed for the design of multiloading panels. Each PCR run was performed in a total volume of 10 μL, containing 50 ng of DNA. Reaction mixtures contained 1x PCR buffer, 2 mM of MgCl_2_, 0.2 μM of each primer, 0.2 mM of dNTPs, and 0.5U of Taq polymerase. The amplification conditions included an initial denaturation step of 95 °C for 5 min, followed by 35 cycles of 95 °C for 30 s, 90 s at 56 °C or 58 °C, 72 °C for 1 min, and a final extension at 72 °C for 30 min. The PCR products were separated by capillary electrophoresis in an automatic ABI Prism 3130XL Genetic Analyzer^®^ (Applied Biosystems, Foster City, CA, USA). Genotyping was performed using GeneMapper v.4.0 software (Applied Biosystems).

### 2.3. Statistical Analysis

The most used genetic diversity parameters, such as the number of alleles per microsatellite, allelic frequencies, mean number of alleles per microsatellite, private alleles, expected and observed heterozygosity (He and Ho, respectively), and polymorphic information content (PIC), were calculated for each of the 13 microsatellite markers, using the CERVUS 3.0.3 [42] and GENETIX 4.0.5 [43] software. Possible deviations from the Hardy–Weinberg equilibrium (HWE), either due to an excess or to a deficit of heterozygous in the total population and within subpopulations, were estimated using Fisher’s exact test implemented in the GENEPOP 4.0.11 software [44]. Furthermore, to guarantee the quality of the results, the null allele frequencies were also calculated using Micro-Checker 2.2.3 [45]. The level of genetic differentiation among individuals, within and between subpopulations, was calculated by the analysis of molecular variance (AMOVA) test using the ARLEQUIN 3.1 software [46]. The extent of genetic differentiation between the two llama subpopulations, Ch’aku and Q’ara, was quantified using the F-statistics (F_IS_, F_IT_, and F_ST_; [47]) using GENEPOP 4.0.11 and corroborated with FSTAT 2.9.3.2 [48]. The effective number of migrants per generation (Nm, [19]) was estimated by GENEPOP 4.0.11.

The genetic structure was determined through a grouping analysis of the individuals in a different number of inferred clusters (K) using an analysis based on the ‘admixture’ ancestry model implemented in the STRUCTURE 2.3 software [49]. The burn-in period was set to 50,000 followed by 500,000 Markov chain Monte Carlo (MCMC) iterations. Independent runs of K were performed from 1 to 7 clusters and were repeated 4 times to check the consistency of the results. Finally, a factorial correspondence analysis was performed with GENETIX 3.0.3 to further assess the genetic relationships between the llama types, describing the association of qualitative variables in which each individual is represented just once for the value of each modality (microsatellites) and variable (alleles per microsatellite).

## 3. Results

### 3.1. Genetic Diversity Assessment

The 13 microsatellites used in this study were polymorphic and revealed a high level of genetic diversity in the population of llamas from the Camelid Germplasm Bank—Quimsachata. The number of alleles, He, Ho, and PIC values for each microsatellite for Ch’aku and Q’ara as separated populations and as a global population are shown in Table 1. 

In total, 157 alleles were found across the 251 individuals, with an average of 12.08 alleles per microsatellite (ranging from 8 for LCA54, LCA83, and LCA85 up to 19 for YWLL08 and YWLL59). The He value was higher than the Ho value, with an average of 0.707 in the global population. Additionally, the He and Ho values were always higher in the Ch’aku subpopulation compared to the Q’ara. The PIC value ranged from 0.517 (LCA54) to 0.883 (YWLL08). 

For the global population, 7 out of the 13 microsatellites departed from the HWE (*p* < 0.05). The inbreeding coefficient (F_IS_) was estimated for each microsatellite for the global population and for the Ch’aku and Q’ara subpopulations, and they were positive and significantly different from zero (*p* > 0.05) (Table 2). Moreover, we observed a deficit of heterozygotes and 7.1% more homozygotes than would be expected under the HWE (Table 2). The existence of null alleles was assessed with Micro-Checker, and only the microsatellites LCA82A, YWLL59A, LCA85A, and GLM4 showed signs of having them (Table S2). On the other hand, a total of 35 private alleles were observed (15 for Ch’aku and 20 for Q’ara subpopulations), but their frequencies were very low (<0.05) (Table 3).

### 3.2. Genetic Differentiation between Ch’aku and Q’ara

The genetic differentiation coefficient value (F_ST_) between the Ch’aku and Q’ara types showed very low genetic differentiation (F_ST_ = 0.01) (Table 4), whereas a high gene flow value was observed (Nm = 30.9 migrants per generation). Additionally, the analysis of molecular variance indicated that the highest variance was due to variation within the populations (93.95%), while only 1.02% variance was observed between the two subpopulations (Table S3). The F_ST_ value calculated by Arlequin 3.1 was 0.0102 and coincided with the value given by FSTAT 2.9.3.2 [48]. 

**Figure 2:**
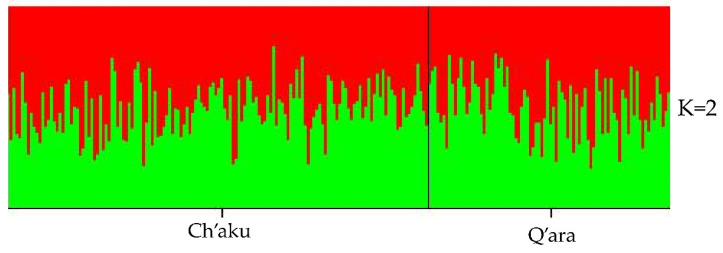
Structure analysis of the global llama population by cluster assignment using the Bayesian analysis (K = 2). The colored bars indicate the probability of assignment to either Cluster 1 (red) or 2 (green). The black line divides the Ch’aku and Q’ara subpopulations.

**Figure 3:**
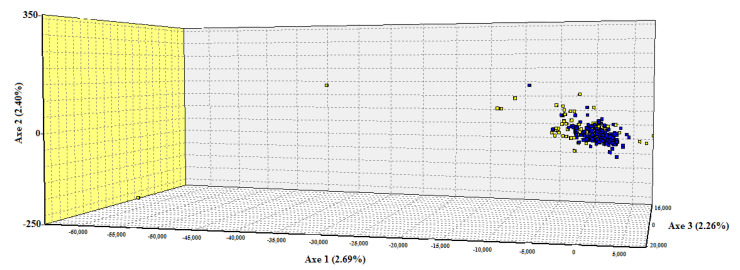
Factorial correspondence analysis between the Ch’aku and Q’ara subpopulations. Yellow, Ch’aku; Blue, Q’ara.

Bayesian analysis carried out by the STRUCTURE software [49], using seven independent runs (K = 1, 2, 3, 4, 5, 6, 7) and each one repeated four times, did not show any evidence of genetic differentiation or population subdivisions. The highest likelihood was obtained when K = 2 and the individuals of both phenotypes were assigned to two clusters, but there was not a clear separation between the two llama subpopulations (Figure 2). Thus, there was no evidence of population structure in the Camelid Germplasm Bank—Quimsachata. Additionally, the correspondence factorial analysis showed a graphic representation of the genetic relationship between the Ch’aku and Q’ara subpopulations (Figure 3). This analysis indicated once again the low genetic differentiation between the subpopulations, given that both overlap with each other and do not form clear independent groups. 

## 4. Discussion

There has been an upsurge in attempts to conserve natural resources, since it is well known that the loss of genetic variability diminishes the ability to recover endangered species [50] and decreases the chance to improve the performance of animals involved in production systems [51]. In this scenario, the Camelid Germplasm Bank—Quimsachata plays a crucial role in conserving a natural, economic, cultural, and historical resource of the SAC populations in Peru. The population of llamas in this Bank must have high genetic diversity to be considered an appropriate genetic stock that could contribute to ensuring its conservation, aid in implementing future strategies to face the loss of diversity, and increase the viability and productivity of llama populations from other regions of Peru. This study is the first to report on the genetic diversity and population structure of the llama population in the Camelid Germplasm Bank—Quimsachata using microsatellite markers.

A representative sample of llama individuals (*n* = 251) was analyzed by using 13 microsatellites specific to the SAC. The results showed a high level of genetic diversity in the population of llamas, with an average of 12.08 alleles per microsatellite and an expected heterozygosity of 0.758. The Camelid Germplasm Bank—Quimsachata keeps the birth and mating records of each individual and that information is used to avoid mating between close relatives [13]. Thus, our results might indicate that this management strategy has contributed to maintaining high genetic variability, and it will be complemented with the routine assessment of the genetic variability by means of molecular markers such as microsatellites or single-nucleotide polymorphisms (SNPs). Furthermore, our results are similar to those from Argentinean (7.33–8.33 mean alleles per microsatellite and He of 0.47–0.9; [1,52]) and Bolivian (12.04 alleles per microsatellite and He = 0.68) llama populations [2]. Likewise, in those studies, the He was always higher than the Ho, and a higher value of He was reported in the Quimsachata llama population compared to the Bolivian llama population. Nonetheless, it is important to mention that they used different sets of microsatellite markers (many of which were individually used in this study), and in the case of Barreta et al. (2013), they were not necessarily specific for SACs. Thus, to make a more adequate comparison, we would ultimately have to use the same set of microsatellites used in those studies. 

All the microsatellites analyzed were highly polymorphic (PIC > 0.5), especially YWLL08 (0.883), YWLL44 (0.863), YWLL59 (0.819), and LCA85 (0.8). Interestingly, we found alleles previously unrecorded in their original studies; for instance, YWLL08, YWLL44, YWLL59, LCA82, LCA83, GLM4, and LGU76 reported 13, 11, 10, 5, 7, 9, and 8 alleles in the first studies, respectively [22,26,27,28,40], whereas we recorded 19, 17, 19, 9, 8, 10, and 15, respectively (Table 1). However, this difference could be due to the distinctive population sample and size used by the initial investigators. 

The F_IS_ media value for the total population (0.063) and the microsatellites showing deviations from the HWE were explained by heterozygote deficiency. The excess of homozygotes in a domestic population indicates loss of genetic variability and could be explained by a lack of random mating, which occurs during the artificial selection of herds [1], population subdivisions (Wahlund effect), gene flow, or the existence of null alleles [53]. Indeed, we observed the presence of null alleles in four microsatellite markers (LCA82A, YWLL59A, LCA85A, and GLM4; Table S2), which could have potentially increased the observed homozygosity value. Additionally, other possible explanations for homozygote excess could be due to the evolutionary history of llamas, consisting of polygynous behavior in which herds contain an α male who controls the access of other males to its territory and expels young males while retaining the female individuals. This behavior of a young male’s exclusion is still present in managed populations [1,54]. A departure from the HWE could be due not only to a genotyping error (null alleles) in some of the microsatellites but also to the repetitive mating of individuals within the same herd and to the low quantity of breeding males.

Regarding the llama population structure from the Camelid Germplasm Bank—Quimsachata, although we found a large number of private alleles in the Ch’aku and Q’ara subpopulations (15 and 20, respectively; Table 3), the AMOVA analysis showed that most of the variation came from the variability within the Ch’aku and Q’ara subpopulations (93.95%), whereas only 1.02% was due to the variability between the subpopulations (Table S3). Furthermore, their genetic differentiation index (F_ST_) was 0.0102 along with a high gene flow value (Nm = 30.5). These values, according to [47], are indicators of low genetic differentiation between the two subpopulations and a weak genetic structure. These results are confirmed by the cluster analysis and the correspondence factorial analysis (Figure 3) and are similar to the values estimated by [2], among and within regional groups of Bolivian llamas.

Importantly, the breeding management of the Bank has been highly controlled since its beginnings, that is, all Ch’aku and Q’ara males are reared together but kept separately from Ch’aku and Q’ara females (who are also reared together) and the males and females of the same phenotype are only brought together for breeding. Therefore, the low genetic structure is most likely a result of their own ancestral domestication process, which involved frequent exchanges of reproductive males and, hence, crossbreeding between both types. For future studies to elucidate a potential genetic differentiation between these phenotypes, we propose the use of genes related to observed phenotypic traits (e.g., the diameter of fiber), which could help to identify allelic variants related to the studied phenotypes [2], or the use of SNPs, which cover the whole llama genome [12].

## 5. Conclusions

This study is the first assessment of the genetic diversity and structure of the Peruvian llama (*L. glama*) population from the Camelid Germplasm Bank—Quimsachata utilizing microsatellites. The set of microsatellites used was highly polymorphic and, hence, can be utilized to track the genetic variability of these animals to avoid a reduction in the effective population size. Overall, our results show that the llama population of the Camelid Germplasm Bank—Quimsachata presents a high level of genetic diversity; thus, it can be considered as an adequate stock for the conservation of this natural resource. However, it would be important to identify other Peruvian llama populations that could help to increase the diversity of the Bank and enable a higher representation of Peruvian llamas.

On the other hand, although bred separately and managed as two distinct subpopulations, we observed low genetic differentiation between the Ch’aku and Q’ara phenotypes. Therefore, additional analysis with genes related to phenotypic observed differences should be carried out to assess potential genetic differentiation between these two phenotypes.

## Figures and Tables

**Table 1:** Genetic diversity parameters of each llama subpopulation and the global population.

Micro Satellite	Ch’aku				Q’ara				Global Population			
	A	Ho	He	PIC	A	Ho	He	PIC	A	Ho	He	PIC
GLM4	9	0.66	0.78	0.75	10	0.76	0.78	0.66	10	0.72	0.78	0.71
LAB1	13	0.72	0.79	0.53	13	0.70	0.73	0.5	14	0.71	0.76	0.52
LCA54	4	0.6	0.61	0.75	8	0.53	0.56	0.75	8	0.56	0.59	0.75
LCA65	8	0.61	0.65	0.82	8	0.61	0.64	0.77	9	0.61	0.64	0.79
LCA77	8	0.66	0.64	0.86	10	0.67	0.68	0.86	11	0.66	0.67	0.86
LCA82	9	0.68	0.79	0.82	7	0.52	0.72	0.81	9	0.57	0.75	0.82
LCA83	8	0.79	0.78	0.77	7	0.72	0.74	0.71	8	0.75	0.76	0.74
LCA85	8	0.76	0.85	0.90	7	0.74	0.81	0.86	8	0.75	0.82	0.88
LGU76	14	0.83	0.84	0.83	14	0.75	0.79	0.78	15	0.78	0.81	0.8
YWLL08	17	0.90	0.91	0.59	17	0.83	0.87	0.63	19	0.86	0.89	0.62
YWLL44	16	0.84	0.89	0.58	17	0.85	0.87	0.59	17	0.85	0.875	0.59
YWLL59	15	0.73	0.84	0.74	17	0.75	0.84	0.70	19	0.74	0.84	0.73
VOLP03	9	0.74	0.68	0.62	8	0.59	0.64	0.57	10	0.64	0.66	0.59
Average	10.5	0.73	0.77	0.74	10.9	0.69	0.74	0.71	12.08	0.71	0.76	0.72

A, allele number; Ho, observed heterozygosity; He, expected heterozygosity; PIC, polymorphic information content.

**Table 2:** Analysis of the Hardy–Weinberg equilibrium for each microsatellite of the global llama population of the Camelid Germplasm Bank—Quimsachata.

Global Population				Ch’aku Population				Q’ara Population			
Microsatellite	Deficit of Heterozygotes (*p*-Value)	Standard Deviation	F_IS_	Microsatellite	Deficit of Heterozygotes (*p*-Value)	Standard Deviation	F_IS_	Microsatellite	Deficit of Heterozygotes (*p*-Value)	Standard Deviation	F_IS_
GLM4	0.0062	0.0014	0.073	GLM4	0.0016	0.0004	0.145	GLM4	0.2851	0.012	0.0335
LAB1	0.0002	0.0002	0.056	LAB1	0	0	0.087	LAB1	0.176	0.0128	0.0383
LCA54	0.0217	0.0051	0.04	LCA54	0.0831	0	0.0211	LCA54	0.0475	0.0054	0.0512
LCA65	0.054	0.0134	0.054	LCA65	0.2489	0.0167	0.0676	LCA65	0.1858	0.0127	0.0467
LCA77	0.259	0.015	0.004	LCA77	0.6392	0.0144	−0.0216	LCA77	0.2113	0.0206	0.0171
LCA82	0.0001	0.0002	0.223	LCA82	0.0142	0.0018	0.1327	LCA82	0.0024	0.0006	0.2788
LCA83	0.0463	0.0066	0.01	LCA83	0.0302	0.0025	−0.0152	LCA83	0.2907	0.0089	0.0251
LCA85	0.0002	0.0001	0.089	LCA85	0.0038	0.0006	0.1191	LCA85	0.0155	0.0013	0.0788
LGU76	0.139	0.0227	0.039	LGU76	0.1939	0.0128	0.0149	LGU76	0.2682	0.0155	0.0537
YWLL08	0.182	0.0458	0.034	YWLL08	0.4779	0.0205	0.0127	YWLL08	0.1105	0.0113	0.0473
YWLL44	0.118	0.0133	0.033	YWLL44	0.1722	0.0133	0.0372	YWLL44	0.2247	0.0153	0.0335
YWLL59	0.0001	0.0001	0.118	YWLL59	0	0	0.1255	YWLL59	0.0373	0.0068	0.109
VOLP03	0.434	0.0511	0.027	VOLP03	0.9412	0.0091	−0.0785	VOLP03	0.1508	0.0113	0.0905

F_IS_, coefficient of inbreeding.

**Table 3:** Private allele size of the 13 analyzed microsatellite markers of the Ch’aku and Q’ara llama subpopulations.

Microsatellite	Ch’aku	Q’ara
GLM4	*	214
LAB1	177	203
LCA54	*	159, 171, 173, 179
LCA65	202	200
LCA77	264	256, 262, 278
LCA82	126, 132	*
LCA83	216	*
LCA85	214	*
LGU76	285	279
YWLL08	181, 201	157, 189
YWLL44	127	133, 143
YWLL59	102, 114	138, 142, 148, 150
VOLP03	153, 171	191
Total	15	20

* No private alleles found.

**Table 4:** F_ST_ values of each microsatellite marker in the global population of llamas from the Camelid Germplasm Bank—Quimsachata.

Microsatellites	F_ST_
GLM4	0.005
LAB1	0.009
LCA54	0.033
LCA65	0.001
LCA77	0.005
LCA82	0.017
LCA83	0.022
LCA85	0.009
LGU76	0.009
YWLL08	0.016
YWLL44	0.005
YWLL59	0.001
VOLP03	0.002
Average	0.01

## Supplementary Materials

The following are available online at https://www.mdpi.com/2073-4425/11/5/541/s1, Table S1: Primer sets used for genotyping the microsatellites, Table S2: Existence and Frequencies of null alleles found in each microsatellite marker analyzed with the software Micro Checker 2.2.3 and CERVUS 3.0.3, respectively Table S3: Analysis of molecular variance (AMOVA) between the subpopulations of llamas Ch’aku and Q’ara.
