# Supplementary material for: Genetic Diversity and Population Structure of Llamas (Lama glama) from the Camelid Germplasm Bank—Quimsachata
Source: Genes (Basel). 2020 May 12;11(5):541. doi: 10.3390/genes11050541 (PMC7291211; doi:10.3390/genes11050541)
Supplement: Supplementary file 1 [file genes-11-00541-s001.pdf]

Supplementary Material
Paredes et al.

Table S1. Primer sets used for genotyping the microsatellites.

Microsatellite	Primer sequence (5'->3')	Fragment size (bp)	Alignment temperature (°C)	Fluorescent dyes	References
Tail M13	TGTAAAAC GACGGCCAGT	18	--		Steffens *et al.*, 1993
GLM4 F	M13-TGAAGGAATGCAGATGAGAAGC	181-205	58	6-FAM	Bustamante *et al.*, 2003
R	TAGCTACAAACTTCCATGACAC				
LAB1 F	M13-AGAGGATCAATCCCTCTGAGAT	161-189	58	NED	Bustamante *et al.*, 2003
R	ATTAGAGGCCAGTATAACAATC				
LCA54 F	M13-CACGTATACTTAAGAAGGG	147-157	58	6-FAM	Penedo *et al.*, 1999a
R	CTGCAAGTTCGGAGAGAAA				
LCA65 F	M13-TTTTTCCCCTGTGGTTGAAT	165-191	58	HEX	Penedo *et al.*, 1999a
R	AACCTCAGCTGTTGTCAGGGG				
LCA77 F	M13TGTTGACTAGAGCCTTTTCTTCTTT	233-263	58	6-FAM	Penedo *et al.*, 1999a
R	GGGCAAGAGAGACTGACTGG				
LCA82 F	M13-CGTGACACCAGGCTAAGTGA	108-124	58	6-FAM	Penedo *et al.*, 1999b
R	TTTCAGATGGTAGCTTTAAAAATT				
LCA83 F	M13-A TTCACCTTGCAGTTCCTGG	191-221	58	HEX	Penedo *et al.*, 1999b
R	GACTCCAAGCAGGACGAGAC				
LCA85 F	M13-CACGTATACAGACCAGAGAAGGG	194-210	58	6-FAM	Penedo *et al.*, 1999b
R	CTGCAAGGGATTACGGAGAA				
LGU76 F	M13-TTCCTTCCATTGAAGCAGGT	233-261	58	6-FAM	Sarno *et al.*, 2000
R	TGAGATGCACTGCTTTGGATA				
YWLL08 F	M13-ATCAAGTTTGAGGTGCTTTCC	126-188	58	6-FAM	Lang *et al.*, 1996
R	CCATGGCATTGTGTTGAAGAC				
YWLL44 F	M13-CTCAACAATGCTAGACCTTGG	81-130	58	HEX	Lang *et al.*, 1996

R	GAGAACACAGGCTGGTGAATA				
YWLL59 F	M13-TGTGCAGGAGTTAGGTGTA	96-136	58	NED	Lang *et al.*, 1996
R	CCATGTCTCTGAAGCTCTGGA				
VOLP03 F	M13-AGACGGTTGGGAAGGTGGTA	129-169	56	NED	Obreque *et al.*, 1998
R	CGACAGCAAGGCACAGGA				

Table S2. Existence and Frequencies of null alleles found in each microsatellite marker analyzed with the software Micro Checker 2.2.3 and CERVUS 3.0.3, respectively.

Locus	Global (F0)	Q'ara	Ch'aku
GLM4	0.035	no	yes
LAB1	0.029	no	no
LCA54	0.028	no	no
LCA65	0.031	no	no
LCA77	-0.003	no	no
LCA82	0.133	yes	yes
LCA83	0.009	no	no
LCA85	0.046	yes	yes
LGU76	0.022	no	no
YWLL08	0.02	no	no
YWLL44	0.016	no	no
YWLL59	0.062	yes	yes
VOLP03	0.016	no	no

*F0, Frequencies of null alleles from the global population

Table S3. Analysis of molecular variance (AMOVA) between the subpopulations of llamas Ch'aku and Q'ara.

Source of variation	Degree of freedom	Sum of Squares	Variance Components	Percentage of Variance (%)
Between populations	1	12.845	0.03837	1.02
Between individuals between populations	249	970.878	0.1886	5.03
Within the population	251	884	3.52191	93.95
